# Supplementary material for: The Association of Variants within Types V and XI Collagen Genes with Knee Joint Laxity Measurements
Source: Genes (Basel). 2022 Dec 14;13(12):2359. doi: 10.3390/genes13122359 (PMC9778334; doi:10.3390/genes13122359)
Supplement: Supplementary file 1 [file genes-13-02359-s001.zip › genes-1916733-supplementary.pdf]

**SUPPLEMENTARY MATERIAL**

**Supplementary Table S1:** The intraclass correlation coefficient (ICC), standard error of measurement (SEM) as well as the minimum detectable change at 90% confidence (MDC90) of genu recurvatum (passive and active) and anterior-posterior laxity (133 N anterior and posterior translation compliance index, active and maximum displacement) measurements in the non-dominant leg.

| | ICC(2,1) | SEM | MDC90 | N |
|---|---|---|---|---|
| **Genu Recurvatum** | | | | |
| ▪ **Passive** (°) | 0.79 | 2.08 | 4.86 | 30 |
| ▪ **Active** (°) | 0.79 | 2.12 | 4.95 | 30 |
| **Anterior-posterior laxity** | | | | |
| ▪ **133 N Anterior Translation** (mm) | 0.80 | 1.05 | 2.46 | 27 |
| ▪ **133 N Posterior Translation** (mm) | 0.61 | 0.68 | 1.59 | 27 |
| ▪ **Compliance Index** (mm) | 0.78 | 0.84 | 1.95 | 28 |
| ▪ **Active Displacement** (mm) | 0.83 | 0.96 | 2.24 | 27 |
| ▪ **Maximum Displacement** (mm) | 0.87 | 0.87 | 2.04 | 27 |

The intra-rater reliability of the knee laxity measurements was determined in a subset of 30 (15 male and 15 female) of the recruited participants.

The average age, height, body mass and BMI of the sub-set of the participants were 28.0 ± 5.7 years, 173.8 ± 9.4 cm, 72.3 ± 12.2 kg and 23.8 ± 2.3 kg/m$^2$, respectively. Twenty-three (76.7%) of the participants were right leg dominant

Anterior-posterior laxity was measured using the measured using the KT-1000 arthrometer

ICC values <0.5 indicate poor reliability, between 0.5 and 0.75 moderate reliability, between 0.75 and 0.9 good reliability, and >0.9 excellent reliability

**Supplementary Table S2:** Descriptive data for the five trials of the reliability study for of genu recurvatum (passive and active) and anterior-posterior laxity (133 N anterior (Ant) and posterior (Post) translation (Trans), compliance index, active and maximum (Max) displacement) measurements in the non-dominant leg.

| | **Trial 1** | **Trial 2** | **Trial 3** | **Trial 4** | **Trial 5** | **N** |
|---|---|---|---|---|---|---|
| **Genu Recurvatum** | | | | | | |
| ▪ **Passive** (°) | 176.6 ± 4.0 | 177.2 ± 4.6 | 177.9 ± 4.0 | 177.2 ± 5.2 | 176.9 ± 5.0 | 30 |
| ▪ **Active** (°) | 174.9 ± 4.0 | 175.0 ± 4.9 | 175.5 ± 4.2 | 175.1 ± 4.6 | 175.4 ± 5.1 | 30 |
| **Anterior-posterior laxity** | | | | | | |
| ▪ **133 N Ant Trans** (mm) | 6.5 ± 2.3 | 6.6 ± 2.3 | 6.7 ± 2.4 | 6.9 ± 2.3 | 6.9 ± 2.3 | 27 |
| ▪ **133 N Post Trans** (mm) | 3.7 ± 1.1 | 3.6 ± 1.1 | 3.6 ± 1.0 | 3.7 ± 0.9 | 3.9 ± 1.4 | 27 |
| ▪ **Compliance Index** (mm) | 4.2 ± 2.0 | 4.1 ± 1.9 | 4.3 ± 2.1 | 4.3 ± 2.0 | 4.3 ± 2.1 | 28 |
| ▪ **Active Displacement** (mm) | 4.6 ± 2.5 | 4.4 ± 2.1 | 4.5 ± 2.4 | 4.4 ± 2.2 | 4.9 ± 2.4 | 27 |
| ▪ **Max Displacement** (mm) | 7.2 ± 2.0 | 7.2 ± 2.5 | 7.1 ± 2.6 | 7.3 ± 2.5 | 7.3 ± 2.4 | 27 |

Variables are reported as average ± standard deviation

Anterior-posterior laxity was measured using the measured using the KT-1000 arthrometer

**Supplementary Table S3:** The *COL5A1* rs12722 (T/C) genotype effects on the general characteristics, Beighton score and sit and reach measurements of participants with a history of an uninjured non-dominant leg.

| | ***COL5A1* rs12722** | | |
|---|---|---|---|
| | **CC** | **CT + TT [a]** | **P-value** |
| | n = 15 | n = 89 | |
| **Sex** (% male) | 66.7 | 53.9 | 0.411 |
| **Age** (years) | 26.0 (24.0; 30.0) | 26.0 (24.0; 30.0) | 0.967 |
| **Height** (cm) | 176.5 ± 11.6 | 174.3 ± 9.7 | 0.439 |
| **Body mass** (kg) | 75.0 (61.2; 88.0) | 71.7 (61.1; 80.7) | 0.541 |
| **BMI** ($kg/m^2$) | 23.9 (22.2; 25.7) | 23.7 (21.1; 25.5) | 0.627 |
| **Flexibility Training** (% Yes) | 53.3 | 55.1 | 1.000 |
| **Beighton Score** | 1.0 (0.0; 2.0) | 2.0 (0.0; 4.0) | 0.077 |
| **Sit and Reach** (cm) | 41.0 (38.4; 47.5) | 42.9 (36.0; 50.8) [b] | 0.901 |

Sex and flexibility training are reported as relative percentages. The remaining continuous variables are reported as average ± standard deviation or median (IQR)

Unable to genotype 2 participants for rs12722

BMI, body mass index

[a] The TT (n = 28) and CT (n = 61) genotypes of rs12722 were combined for the analysis because of the previous association of the CC genotype with range of motion (Brown et al. 2011; Lim et al. 2015)

[b] n = 85

**Supplementary Table S4:** The *COL11A1* rs3753841 (T/C), rs1676486 (C/T) and *COL11A2* rs1799907 (A/T) genotype effects on the general characteristics, Beighton score and sit and reach measurements of participants with a history of an uninjured non-dominant leg.

| | ***COL11A1* rs3753841** | | | ***COL11A1* rs1676486** | | | ***COL11A2* rs1799907** | | |
|---|---|---|---|---|---|---|---|---|---|
| | **CC** | **TC + TT** [a] | **P-value** | **TT** | **CT + CC** [b] | **P-value** | **AA** | **AT + TT** [c] | **P-value** |
| | n = 17 | n = 89 | | n = 5 | n = 101 | | n = 49 | n = 55 | |
| **Sex** (% male) | 58.8 | 53.9 | 0.794 | 80.0 | 53.5 | 0.374 | 53.1 | 56.4 | 0.844 |
| **Age** (years) | 25.0 (23.0; 27.0) | 26.0 (24.0; 30.0) | 0.202 | 25.0 (23.5; 28.0) | 26.0 (24.0; 30.0) | 0.549 | 26.0 (23.0; 30.0) | 26.0 (24.0; 30.0) | 0.559 |
| **Height** (cm) | 173.7 ± 11.3 | 174.9 ± 9.6 | 0.656 | 173.5 ± 10.0 | 174.7 ± 9.9 | 0.788 | 173.7 ± 9.1 | 175.9 ± 10.6 | 0.253 |
| **Body mass** (kg) | 67.0 (60.7; 79.5) | 73.3 (61.0; 81.1) | 0.532 | 75.2 (63.1; 79.5) | 71.7 (60.8; 81.1) | 0.837 | 67.7 (60.6; 78.8) | 75.0 (61.2; 82.0) | 0.202 |
| **BMI** ($kg/m^2$) | 23.7 (21.0; 24.3) | 23.7 (21.3; 25.6) | 0.595 | 24.3 (22.2; 25.6) | 23.7 (21.1; 25.5) | 0.551 | 23.3 (21.1; 25.5) | 23.7 (21.6; 25.5) | 0.594 |
| **Flexibility training** (% Yes) | 70.6 | 52.8 | 0.111 | 60.0 | 55.4 | 1.000 | 51.0 | 58.2 | 0.555 |
| **Beighton Score** | 1.0 (0.0; 4.0) | 2.0 (0.0; 4.0) | 0.588 | 3.0 (0.0; 4.0) | 2.0 (0.0; 4.0) | 0.983 | 2.0 (0.0; 4.0) | 1.0 (0.0; 4.0) | 0.591 |
| **Sit and Reach** (cm) | 46.4 ± 9.7 [d] | 42.5 ± 10.6 [e] | 0.172 | 43.3 ± 10.0 | 43.1 ± 10.6 [f] | 0.973 | 42.2 ± 10.1 [g] | 44.1 ± 11.1 [h] | 0.378 |

Sex and flexibility training are reported as relative percentages. The remaining continuous variables are reported as average ± standard deviation or median (IQR)

Unable to genotype 2 participants for rs1799907

BMI, body mass index

Similar to the *COL5A1* analysis, the *COL11A1* and *COL11A2* genotypes containing the allele associated with increased risk of musculoskeletal soft tissue injury were combined and compared to the protective CC, TT and AA genotypes for rs3753841, rs1676486 and rs1799907 respectively (Hay et al. 2013)

[a] *COL11A1* rs3753841: TC = 49 and TT = 40; [b] *COL11A1* rs1676486: CT = 28 and CC = 73 [c] *COL11A2* rs1799907: AT = 43 and TT =12

[d] n = 16, [e] n = 87, [f] n = 98, [g] n = 48, [h] n = 53

**Supplementary Table S5:** The *COL5A1* rs12722 (T/C) genotype effects on the general characteristics, Beighton score and sit and reach measurements for the subset of participants with a history of an uninjured non-dominant leg measured on the Robotic Knee Testing (RKT) device.

| | ***COL5A1* rs12722** | | |
|---|---|---|---|
| | **CC** | **CT + TT** [a] | **P-value** |
| | n = 11 | n = 65 | |
| **Sex** (% male) | 72.7 | 58.5 | 0.511 |
| **Age** (years) | 26.0 (22.0; 33.0) | 26.0 (24.0; 30.0) | 0.988 |
| **Height** (cm) | 180.1 ± 10.5 | 174.4 ± 9.5 | 0.061 |
| **Body mass** (kg) | 77.6 ± 13.0 | 73.6 ± 12.8 | 0.341 |
| **BMI** ($kg/m^2$) | 24.0 (22.2; 25.7) | 23.8 (22.4; 26.0) | 0.912 |
| **Flexibility Training** (% Yes) | 54.5 | 56.9 | 1.000 |
| **Beighton Score** | 1.0 (0.0; 3.0) | 2.0 (0.0; 4.0) | 0.176 |
| **Sit and Reach** (cm) | 44.8 ± 10.1 | 43.4 ± 10.4 [b] | 0.690 |

Sex and flexibility training are reported as relative percentages. The remaining continuous variables are reported as average ± standard deviation or median (IQR)

Unable to genotype 2 participants for rs12722

BMI, body mass index

[a] The TT (n = 17) and CT (n = 48) genotypes of rs12722 were combined for the analysis because of the previous association of the CC genotype with range of motion (Brown et al. 2011; Lim et al. 2015)

[b] n = 62

**Supplementary Table S6:** The *COL11A1* rs3753841 (T/C) and *COL11A2* rs1799907 (A/T) genotype effects on the general characteristics, Beighton score and sit and reach measurements for the subset of participants with a history of an uninjured non-dominant leg measured on the Robotic Knee Testing (RKT) device.

| | ***COL11A1* rs3753841** | | | ***COL11A2* rs1799907** | | |
|---|---|---|---|---|---|---|
| | **CC** | **TC + TT** [a] | **P-value** | **AA** | **AT + TT** [b] | **P-value** |
| | n = 11 | n = 66 | | n = 35 | n = 42 | |
| **Sex** (% male) | 54.5 | 60.6 | 0.748 | 57.1 | 61.9 | 0.816 |
| **Age** (years) | 25.0 (23.0; 28.0) | 26.5 (24.0; 31.3) | 0.260 | 26.0 (24.0; 31.0) | 26.5 (23.8; 30.0) | 0.805 |
| **Height** (cm) | 174.7 ± 13.1 | 175.0 ± 9.3 | 0.915 | 174.0 ± 9.2 | 175.8 ± 10.2 | 0.433 |
| **Body mass** (kg) | 70.2 ± 12.5 | 74.6 ± 12.9 | 0.289 | 71.9 ± 11.4 | 75.7 ± 13.8 | 0.199 |
| **BMI** ($kg/m^2$) | 23.7 (21.0; 24.3) | 24.0 (22.4; 26.0) | 0.122 | 23.8 (21.2; 25.6) | 23.9 (22.6; 26.0) | 0.631 |
| **Flexibility training** (% Yes) | 63.6 | 56.1 | 0.749 | 57.1 | 57.1 | 1.000 |
| **Beighton Score** | 1.0 (0.0; 3.0) | 2.0 (0.0; 4.0) | 0.317 | 2.0 (0.0; 4.0) | 1.0 (0.0; 4.0) | 0.522 |
| **Sit and Reach** (cm) | 46.2 ± 10.6 [c] | 43.3 ± 10.2 [d] | 0.404 | 42.4 ± 10.7 [e] | 44.9 ± 9.9 [f] | 0.299 |

Sex and flexibility training are reported as relative percentages. The remaining continuous variables are reported as average ± standard deviation or median (IQR)

Unable to genotype 2 participants for rs1799907

BMI, body mass index

The COL11A1 rs1676486 polymorphisms was not included because there were only 3 particiopants with a TT genotype (15 and 59 participants had TC and CC genotypes respectively)

Similar to the *COL5A1* analysis, the *COL11A1* and *COL11A2* genotypes containing the allele associated with increased risk of musculoskeletal soft tissue injury were combined and compared to the protective CC and AA genotypes for rs3753841 and rs1799907 respectively. (Hay et al. 2013)

[a] *COL11A1* rs3753841: TC = 36 and TT = 30; [b] *COL11A2* rs1799907: AT = 32 and TT =10

[c] n = 10, [d] n = 64, [e] n = 34, [f] n = 40

**Supplementary Table S7:** The *COL5A1* rs12722 (T/C) genotype effects on the (i) passive and active genu recurvatum measurements, (ii) anterior (Ant) and posterior (Post) tibial translation, active and maximum (Max) displacement as well as compliance index measured using the KT-1000 arthrometer, and (iii) internal and external rotation, as well as slack measured using the Robotic Knee Testing (RKT) device of participants with a history of an uninjured non-dominant leg.

| | ***COL5A1* rs12722** | | |
|---|---|---|---|
| | **CC** | **CT + TT** | **P-value** |
| **Genu Recurvatum** [a] | n = 15 | n = 89 | |
| ▪ **Passive** (°) | 178.4 ± 6.8 | 176.6 ± 5.6 | 0.280 |
| ▪ **Active** (°) | 176.4 ± 6.2 | 174.5 ± 5.6 | 0.225 |
| **Anterior-Posterior Tibial Translation** | n = 15 | n = 89 | |
| ▪ **133 N Ant Translation** (mm) | 6.4 ± 2.1 | 7.4 ± 2.7 | 0.189 |
| ▪ **133 N Post Translation** (mm) | 4.5 (3.0; 5.5) | 4.0 (3.0; 5.3) [b] | 0.569 |
| ▪ **Compliance Index** (mm) | 4.2 ± 1.5 | 4.9 ± 2.1 | 0.198 |
| ▪ **Active Displacement** (mm) | 5.2 ± 3.3 | 4.9 ± 2.6 [b] | 0.765 |
| ▪ **Max Displacement** (mm) | 7.0 (6.0; 9.0) | 7.5 (5.5; 10.0) | 0.534 |
| **External-Internal Rotation** | n = 11 | n = 65 | |
| ▪ **External Rotation** (°) | 4.6 ± 1.5 | 5.6 ± 1.1 | **0.017** |
| ▪ **Internal Rotation** (°) | 5.3 (4.8; 6.2) | 5.7 (5.2; 6.4) | 0.130 |
| ▪ **Slack** (°) | 15.9 ± 3.5 | 17.8 ± 3.2 | 0.081 |

Values are expressed as either average ± standard deviation or median (IQR)

Unable to genotype 2 participants for rs12722

Compliance index = 133 N - 67 N Anterior Translation

The TT and CT genotypes of rs12722 were combined for the analysis because of the previous association of the CC genotype and T allele with range of motion (Brown et al. 2011; Lim et al. 2015)

[a] Lower degree values for genu recurvatum indicates a greater amount of knee hyperextension

[b] n = 88

**Supplementary Table S8:** The *COL11A1* rs3753841 (T/C), rs1676486 (C/T) and *COL11A2* rs1799907 (A/T) genotype effects on the (i) passive and active genu recurvatum measurements, (ii) anterior (Ant) and posterior (Post) tibial translation, active and maximum (Max) displacement as well as compliance index measured using the KT-1000 arthrometer, and (iii) internal and external rotation, as well as slack measured using the Robotic Knee Testing (RKT) device of participants with a history of an uninjured non-dominant leg.

| | ***COL11A1* rs3753841** | | | ***COL11A1* rs1676486** | | | ***COL11A2* rs1799907** | | |
|---|---|---|---|---|---|---|---|---|---|
| | **CC** | **TC + TT** | **P-value** | **TT** | **CT + CC** | **P-value** | **AA** | **AT + TT** | **P-value** |
| **Genu Recurvatum** [a] | n = 17 | n = 89 | | n = 5 | n = 101 | | n = 49 | n = 55 | |
| ▪ **Passive** (°) | 177.9 ± 4.3 | 176.7 ± 6.9 | 0.448 | 176.4 ± 5.3 | 176.9 ± 5.8 | 0.842 | 178.0 ± 6.2 | 176.0 ± 5.3 | 0.080 |
| ▪ **Active** (°) | 175.6 ± 4.5 | 174.6 ± 5.8 | 0.490 | 173.7 ± 5.3 | 174.8 ± 5.7 | 0.674 | 175.6 ± 6.1 | 174.0 ± 5.2 | 0.144 |
| **Ant-Post Translation** | n = 17 | n = 89 | | n = 5 | n = 101 | | n = 49 | n = 55 | |
| ▪ **133 N Ant Translation** (mm) | 6.5 (5.8; 8.0) | 7.0 (5.8; 9.0) | 0.600 | 8.1 ± 2.1 | 7.2 ± 2.6 | 0.441 | 7.0 ± 2.3 | 7.5 ± 2.9 | 0.372 |
| ▪ **133 N Post Translation** (mm) | 4.0 (2.6; 5.8) | 4.0 (3.0; 5.3) | 0.991 | 5.0 (3.5; 6.0) | 4.0 (3.0; 5.0) [c] | 0.301 | 4.0 (3.0; 6.0) | 4.0 (3.0; 5.0) [e] | 0.782 |
| ▪ **Compliance Index** (mm) | 5.0 ± 2.4 | 4.7 ± 2.0 | 0.580 | 6.0 (5.0; 7.3) | 4.5 (3.0; 6.3) | 0.071 | 4.6 ± 1.9 | 5.0 ± 2.2 | 0.337 |
| ▪ **Active Displacement** (mm) | 5.0 (2.8; 7.0) | 5.0 (3.0; 7.0) [b] | 0.864 | 6.0 (3.8; 7.0) | 5.0 (3.0; 7.0) [c] | 0.558 | 4.7 ± 2.6 [d] | 5.2 ± 2.8 | 0.352 |
| ▪ **Max Displacement** (mm) | 7.0 (4.5; 8.8) | 7.0 (5.8; 10.0) | 0.388 | 8.5 (7.0; 11.0) | 7.0 (5.3; 10.0) | 0.260 | 7.0 (5.3; 10.0) | 7.5 (6.0; 10.5) | 0.442 |
| **External-internal Rotation** | n = 11 | n = 66 | | n = 3 | n = 74 | | n = 35 | n = 42 | |
| ▪ **External Rotation** (°) | 5.8 ± 1.4 | 5.4 ± 1.2 | 0.252 | n.d. | n.d. | n.d. | 5.2 (4.6; 6.5) | 5.4 (4.6; 6.3) | 0.827 |
| ▪ **Internal Rotation** (°) | 6.2 (5.3; 7.0) | 5.5 (5.0; 6.3) | 0.140 | n.d | n.d | n.d | 5.5 (5.0; 6.3) | 5.8 (5.2; 6.4) | 0.417 |
| ▪ **Slack** (°) | 18.4 ± 3.4 | 17.4 ± 3.2 | 0.342 | n.d | n.d | n.d | 16.7 (15.5; 18.7) | 17.9 (15.2; 20.1) | 0.780 |

Values are expressed as either average ± standard deviation or median (IQR)
Unable to genotype 2 participants for rs1799907;
n.d., not determined because only 3 particiopants had a COL11A1 rs1676486 TT genotype
Compliance index = 133 N - 67 N Anterior Translation
Similar to the *COL5A1* analysis, the *COL11A1* and *COL11A2* genotypes containing the allele associated with increased risk of musculoskeletal soft tissue injury were combined and compared to the protective CC, TT and AA genotypes for rs3753841, rs1676486 and rs1799907 respectively (Hay et al. 2013)
[a] Lower degree values for genu recurvatum indicates a greater amount of hyperextension
[b] n = 88, [c] n = 100, [d] n = 48, [e] n = 54

**Supplementary Table S9:** The *COL5A1* rs12722 (T/C) genotype effects on the general characteristics, Beighton score and sit and reach measurements of participants with a history of an uninjured non-dominant leg, as well as those with a history of non-dominant lower limb injury and/or treatment.

| | *COL5A1* rs12722 | | |
|---|---|---|---|
| | **CC** | **CT + TT** [a] | **P-value** |
| | n = 19 | n = 113 | |
| **Sex** (% male) | 68.4 | 57.5 | 0.455 |
| **Age** (years) | 26.0 (24.0; 30.0) | 27.0 (24.0; 32.5) | 0.511 |
| **Height** (cm) | 176.3 ± 10.7 | 174.1 ± 9.6 | 0.364 |
| **Body mass** (kg) | 75.0 (61.9; 86.7) | 73.5 (61.1; 81.4) | 0.583 |
| **BMI** ($kg/m^2$) | 24.0 (22.8; 25.6) | 23.7 (21.5; 26.0) | 0.637 |
| **Flexibility Training** (% Yes) | 57.9 | 55.8 | 1.000 |
| **Beighton Score** | 0.0 (0.0; 2.0) | 2.0 (0.0; 4.0) | 0.088 |
| **Sit and Reach** (cm) | 43.1 ± 11.2 | 42.5 ± 10.8 [b] | 0.814 |

Sex and flexibility training are reported as relative percentages. The remaining continuous variables are reported as average ± standard deviation or median (IQR)

Unable to genotype 2 participants for rs12722

BMI, body mass index

[a] The TT (n = 32) and CT (n = 81) genotypes of rs12722 were combined for the analysis because of the previous association of the CC genotype with range of motion (Brown et al. 2011; Lim et al. 2015)

[b] n = 109

**Supplementary Table S10:** The *COL5A1* rs12722 (T/C) genotype effects on the (i) passive and active genu recurvatum measurements, and (ii) anterior (Ant) and posterior (Post) tibial translation, active and maximum (Max) displacement as well as compliance index measured using the KT-1000 arthrometer of participants with a history of an uninjured non-dominant leg, as well as those with a history of non-dominant lower limb injury and/or treatment.

| | ***COL5A1* rs12722** | | |
|---|---|---|---|
| | **CC** | **CT + TT** [a] | **P-value** |
| **Genu Recurvatum** [b] | n = 19 | n = 113 | |
| ▪ **Passive** (°) | 179.2 ± 6.5 | 176.7 ± 5.4 | 0.075 |
| ▪ **Active** (°) | 176.9 ± 5.8 | 174.5 ± 5.3 | 0.070 |
| **Anterior-Posterior Tibial Translation** | n = 19 | n = 113 | |
| ▪ **133 N Ant Translation** (mm) | 6.7 ± 2.1 | 7.5 ± 2.7 | 0.255 |
| ▪ **133 N Post Translation** (mm) | 4.0 (3.0; 5.0) | 4.0 (3.0; 5.5) [c] | 0.908 |
| ▪ **Compliance Index** (mm) | 4.5 (4.0; 6.0) | 4.5 (3.5; 7.0) | 0.394 |
| ▪ **Active Displacement** (mm) | 5.5 (2.5; 7.0) | 5.0 (3.0; 7.0) [c] | 0.939 |
| ▪ **Max Displacement** (mm) | 7.0 (6.0; 9.0) | 8.0 (5.5; 10.5) | 0.815 |

Values are expressed as either average ± standard deviation or median (IQR)

Unable to genotype 2 participants for rs12722

Compliance index = 133 N - 67 N Anterior Translation

Internal and external rotation, as well as slack were not measured in the participants with a history of non-domiant lower limb injury and/or treatment using the Robotic Knee Testing (RKT) device

[a] The TT (n = 32) and CT (n = 81) genotypes of rs12722 were combined for the analysis because of the previous association of the CC genotype and T allele with range of motion (Brown et al. 2011; Lim et al. 2015)

[b] Lower degree values for genu recurvatum indicates a greater amount of hyperextension

[c] n = 112

**Supplementary Table S11:** The *COL11A1* rs3753841 (T/C), rs1676486 (C/T) and *COL11A2* rs1799907 (A/T) genotype effects on the general characteristics, Beighton score and sit and reach measurements of participants with a history of an uninjured non-dominant leg, as well as those with a history of non-dominant lower limb injury and/or treatment.

| | ***COL11A1* rs3753841** | | | ***COL11A1* rs1676486** | | | ***COL11A2* rs1799907** | | |
|---|---|---|---|---|---|---|---|---|---|
| | **CC** | **TC + TT** [a] | **P-value** | **TT** | **CT + CC** [b] | **P-value** | **AA** | **AT + TT** [c] | **P-value** |
| | n = 19 | n = 114 | | n = 7 | n = 126 | | n = 61 | n = 71 | |
| **Sex** (% male) | 63.2 | 57.0 | 0.803 | 85.7 | 56.3 | 0.238 | 55.7 | 60.6 | 0.194 |
| **Age** (years) | 25.0 (23.0; 28.0) | 27.0 (24.0; 33.0) | 0.073 | 26.0 (24.0; 28.0) | 27.0 (24.0; 33.0) | 0.420 | 26.0 (23.5; 33.0) | 27.0 (24.0; 30.0) | 0.930 |
| **Height** (cm) | 174.5 ± 11.0 | 174.6 ± 9.5 | 0.990 | 175.8 ± 9.1 | 174.5 ± 9.8 | 0.728 | 173.7 ± 9.3 | 175.4 ± 10.1 | 0.316 |
| **Body mass** (kg) | 71.0 (61.4; 79.5) | 73.7 (61.1; 82.1) | 0.652 | 75.6 (64.9; 79.5) | 73.4 (61.1; 81.6) | 0.562 | 71.0 (60.6; 80.8) | 75.0 (62.3; 82.4) | 0.284 |
| **BMI** ($kg/m^2$) | 23.3 ± 2.1 | 24.0 ± 3.2 | 0.401 | 24.3 (23.6; 25.6) | 23.7 (21.6; 25.7) | 0.568 | 23.5 (21.1; 25.7) | 23.8 (22.0; 25.6) | 0.598 |
| **Flexibility training** (% Yes) | 63.2 | 56.1 | 0.625 | 42.9 | 57.9 | 0.461 | 50.8 | 60.6 | 0.294 |
| **Beighton Score** | 1.0 (0.0; 4.0) | 1.5 (0.0; 4.0) | 0.387 | 0.0 (0.0; 4.0) | 1.0 (0.0; 4.0) | 0.421 | 2.0 (0.0; 4.0) | 1.0 (0.0; 4.0) | 0.456 |
| **Sit and Reach** (cm) | 44.6 ± 11.4 [d] | 42.5 ± 10.7 [e] | 0.444 | 39.3 ± 12.5 | 42.9 ± 10.7 [f] | 0.392 | 41.5 ± 10.1 [g] | 43.3 ± 11.5 [h] | 0.532 |

Sex and flexibility training are reported as relative percentages. The remaining continuous variables are reported as average ± standard deviation or median (IQR)
Unable to genotype 1 participant for rs3753841, 1 participant for rs1676486 and 2 participants for rs1799907
BMI, body mass index
Similar to the *COL5A1* analysis, the *COL11A1* and *COL11A2* genotypes containing the allele associated with increased risk of musculoskeletal soft tissue injury were combined and compared to the protective CC, TT and AA genotypes for rs3753841, rs1676486 and rs1799907 respectively (Hay et al. 2013)
[a] *COL11A1* rs3753841: TC = 62 and TT = 52; [b] *COL11A1* rs1676486: CT = 33 and CC = 93 [c] *COL11A2* rs1799907: AT = 56 and TT =15
[d] n = 18, [e] n = 111, [f] n = 122, [g] n = 60, [h] n = 68

**Supplementary Table S12:** The *COL11A1* rs3753841 (T/C), rs1676486 (C/T) and *COL11A2* rs1799907 (A/T) genotype effects on the (i) passive and active genu recurvatum measurements, and (ii) anterior (Ant) and posterior (Post) tibial translation, active and maximum (Max) displacement as well as compliance index measured using the KT-1000 arthrometer of participants with a history of an uninjured non-dominant leg, as well as those with a history of non-dominant lower limb injury and/or treatment.

| | ***COL11A1* rs3753841** | | | ***COL11A1* rs1676486** | | | ***COL11A2* rs1799907** | | |
|---|---|---|---|---|---|---|---|---|---|
| | **CC** | **TC + TT** | **P-value** | **TT** | **CT + CC** | **P-value** | **AA** | **AT + TT** | **P-value** |
| **Genu Recurvatum** [a] | n = 19 | n = 114 | | n = 7 | n = 126 | | n = 61 | n = 71 | |
| ▪ **Passive** (°) | 177.8 ± 4.1 | 176.8 ± 5.8 | 0.473 | 176.9 ± 4.7 | 177.0 ± 5.7 | 0.951 | 177.8 ± 6.2 | 176.4 ± 5.1 | 0.154 |
| ▪ **Active** (°) | 175.7 ± 4.3 | 174.7 ± 5.5 | 0.470 | 174.4 ± 4.9 | 174.8 ± 5.4 | 0.841 | 175.5 ± 6.0 | 174.4 ± 4.9 | 0.252 |
| **Ant-Post Translation** | n = 19 | n = 114 | | n = 7 | n = 126 | | n = 61 | n = 71 | |
| ▪ **133 N Ant Translation** (mm) | 7.0 (6.0; 8.0) | 7.0 (5.5; 9.0) | 0.920 | 8.0 (7.0; 10.0) | 7.0 (5.5; 9.0) | 0.207 | 7.0 (6.0; 9.0) | 7.0 (5.5; 9.5) | 0.749 |
| ▪ **133 N Post Translation** (mm) | 4.0 (2.9; 5.1) [b] | 4.0 (3.0; 5.5) | 0.803 | 5.0 (3.0; 6.0) | 4.0 (3.0; 5.5) [d] | 0.359 | 4.0 (3.0; 5.8) | 4.0 (3.0; 5.1) [f] | 0.878 |
| ▪ **Compliance Index** (mm) | 5.0 (4.0; 6.5) | 4.9 ± 2.2 | 0.714 | 6.0 (5.0; 7.0) | 4.5 (3.4; 6.5) | 0.099 | 4.5 (3.5; 6.0) | 5.0 (3.0; 6.5) | 0.636 |
| ▪ **Active Displacement** (mm) | 4.6 ± 2.1 | 5.0 (3.0; 7.0) [c] | 0.442 | 6.0 (2.5; 7.0) | 5.0 (3.0; 7.0) [d] | 0.934 | 4.8 ± 2.7 [e] | 5.0 ± 2.4 | 0.463 |
| ▪ **Max Displacement** (mm) | 8.0 (5.0; 9.0) | 7.5 (6.0; 10.0) | 0.568 | 9.0 (8.0; 11.0) | 7.3 (5.5; 10.0) | 0.135 | 7.0 (5.8; 10.0) | 8.0 (6.0; 10.5) | 0.496 |

Values are expressed as either average ± standard deviation or median (IQR)
Unable to genotype 1 participant for rs3753841, 1 participant for rs1676486 and 2 participants for rs1799907
Compliance index = 133 N - 67 N Anterior Translation
Internal and external rotation, as well as slack were not measured in the participants with a history of non-domiant lower limb injury and/or treatment using the Robotic Knee Testing (RKT) device
Similar to the *COL5A1* analysis, the *COL11A1* and *COL11A2* genotypes containing the allele associated with increased risk of musculoskeletal soft tissue injury were combined and compared to the protective CC, TT and AA genotypes for rs3753841, rs1676486 and rs1799907 respectively (Hay et al. 2013)
[a] Lower degree values for genu recurvatum indicates a greater amount of hyperextension
[b] n = 18, [c] n = 113, [d] n = 125, [e] n = 60, [f] n = 70

**Supplementary Table S13:** The *COL5A1* rs12722 (T/C), *COL11A1* rs3753841 (T/C), *COL11A1* rs1676486 (T/C) and *COL11A2* rs1799907 (A/T) genotype score effects on the general participant characteristics, Beighton score and sit and reach measurements.

| | Genotype Score | | | |
|---|---|---|---|---|
| | **0** | **2** | **4 or 6** | **P-value** |
| | n = 41 | n = 44 | n = 17 | |
| **Sex** (% male) | 53.7 | 56.8 | 58.8 | 0.924 |
| **Age** (years) | 26.0 (24.0; 30.0) | 26.0 (23.0; 30.0) | 25.0 (23.5; 30.0) | 0.779 |
| **Height** (cm) | 175.5 ± 10.8 | 173.4 ± 8.7 | 176.4 ± 11.2 | 0.734 |
| **Body mass** (kg) | 76.9 (61.3; 82.7) | 69.4 (61.0; 80.7) | 67.1 (60.7; 79.5) | 0.486 |
| **BMI** ($kg/m^2$) | 23.8 (21.5; 26.0) | 23.3 (21.8; 25.5) | 23.4 (21.0; 24.3) | 0.535 |
| **Flexibility Training** (% Yes) | 58.5 | 50.0 | 52.9 | 0.730 |
| **Beighton Score** | 2.0 (0.0; 4.0) | 1.0 (0.0; 4.0) | 2.0 (0.0; 3.5) | 0.668 |
| **Sit and Reach** (cm) | 43.2 ± 12.1 [a] | 43.2 ± 9.8 [b] | 42.6 ± 10.0 | 0.979 |

Sex and flexibility training are reported as percentages, while the the remaining continuous variables are reported as average ± standard deviation or median (IQR)

Participants received a genotype score of 2 for each *COL5A1* rs12722 CC, *COL11A1* rs3753841 CC, *COL11A1* rs1676486 TT and *COL12A1* rs1799907 AA genotypes and 0 for all other genotypes, so that the genotype scores ranged from 0 to 8

None of the participants had a genotype score of 8 and the genotype scores of 4 (n = 13) and 6 (n = 4) combined for the analysis

BMI, body mass index

[a] n = 40; [b] n = 42

**Supplementary Table S14:** The *COL5A1* rs12722 (T/C), *COL11A1* rs3753841 (T/C), *COL11A1* rs1676486 (T/C) and *COL11A2* rs1799907 (A/T) genotype score effects on the general participant characteristics, Beighton score and sit and reach measurements for the subset group of participants measured on the Robotic Knee Testing (RKT) device.

| | **Genotype Score** | | | |
|---|---|---|---|---|
| | **0** | **2** | **4 or 6** | **P-value** |
| | n = 31 | n = 33 | n = 12 | |
| **Sex** (% male) | 61.3 | 57.8 | 66.7 | 0.855 |
| **Age** (years) | 26.0 (24.0; 33.0) | 26.0 (23.0; 30.0) | 25.5 (24.0; 32.3) | 0.887 |
| **Height** (cm) | 175.4 ± 10.0 | 173.2 ± 9.2 | 178.8 ± 11.0 | 0.235 |
| **Body mass** (kg) | 77.1 ± 13.7 | 71.6 ± 12.0 | 74.0 ± 11.8 | 0.225 |
| **BMI** ($kg/m^2$) | 24.0 (21.0; 25.3) | 23.7 (22.3; 25.5) | 23.9 (21.0; 25.3) | 0.247 |
| **Flexibility Training** (% Yes) | 61.3 | 51.5 | 58.3 | 0.726 |
| **Beighton Score** | 2.0 (0.0; 4.0) | 2.0 (0.0; 4.0) | 1.5 (0.0; 3.0) | 0.559 |
| **Sit and Reach** (cm) | 44.5 ± 10.5 [a] | 43.1 ± 10.3 [b] | 42.7 ± 10.3 | 0.817 |

Sex and flexibility training are reported as percentages, while the the remaining continuous variables are reported as average ± standard deviation or median (IQR)

Participants received a genotype score of 2 for each *COL5A1* rs12722 CC, *COL11A1* rs3753841 CC, *COL11A1* rs1676486 TT and *COL12A1* rs1799907 AA genotypes and 0 for all other genotypes, so that the genotype scores ranged from 0 to 8

None of the participants had a genotype score of 8 and the genotype scores of 4 (n = 10) and 6 (n = 2) combined for the analysis

BMI, body mass index

[a] n = 30; [b] n = 31

**Supplementary Table S15:** The *COL5A1* rs12722 (T/C), *COL11A1* rs3753841 (T/C), *COL11A1* rs1676486 (T/C) and *COL11A2* rs1799907 (A/T) genotype score effects on the (i) anterior (Ant) and posterior (Post) tibial translation, active and maximum (Max) displacement as well as compliance index measured using the KT-1000 arthrometer, and (ii) internal and external rotation, as well as slack measured using the Robotic Knee Testing (RKT) device of the participants' non-dominant leg.

| | **Genotype Score** | | | **Global P-value** | **Mutiple Comparisons P-value** |
|---|---|---|---|---|---|
| | **0** | **2** | **4 or 6** | | |
| **Anterior-Posterior Tibial Translation** | n = 41 | n = 44 | n = 17 | | |
| ▪ **133 N Ant Translation** (mm) | 7.6 ± 2.7 | 7.2 ± 2.7 | 6.8 ± 2.0 | 0.540 | 0.269 [a] |
| ▪ **133 N Post Translation** (mm) | 4.0 (3.0; 5.0) | 4.0 (3.0; 6.0) [c] | 4.0 (2.8; 5.0) | 0.788 | 1.000 [b] |
| ▪ **Compliance Index** (mm) | 5.0 ± 2.1 | 4.7 ± 2.1 | 4.5 ± 1.8 | 0.678 | 0.385 [a] |
| ▪ **Active Displacement** (mm) | 5.1 ± 2.6 | 4.9 ± 3.1 [c] | 4.8 ± 2.4 | 0.885 | 0.623 [a] |
| ▪ **Max Displacement** (mm) | 7.5 (5.0; 10.5) | 8.0 (5.6; 10.0) | 7.0 (4.5; 9.0) | 0.533 | 0.584 [b] |
| **External-Internal Rotation** | n=31 | n = 33 | n = 12 | | |
| ▪ **External Rotation** (°) | 5.3 ± 0.9 | 5.5 ± 1.4 | 5.4 ± 1.3 | 0.787 | 0.682 [a] |
| ▪ **Internal Rotation** (°) | 5.8 (5.2; 6.2) | 5.5 (5.0; 6.6) | 5.4 (5.2; 6.6) | 0.959 | 1.000 [b] |
| ▪ **Slack** (°) | 17.5 ± 3.0 | 17.6 ± 3.6 | 17.2 ± 3.3 | 0.943 | 0.855 [a] |

Variables are reported as average ± standard deviation or median (IQR)

Participants received a genotype score of 2 for each *COL5A1* rs12722 CC, *COL11A1* rs3753841 CC, *COL11A1* rs1676486 TT and *COL12A1* rs1799907 AA genotypes and 0 for all other genotypes, so that the genotype scores ranged from 0 to 8

None of the participants had a genotype score of 8 and the genotype scores of 4 and 6 combined for the analysis

Compliance index = 133 N - 67 N Anterior Translation

[a] Test for linear trend

[b] Dunn's multiple comparisons test respectively of the 0 versus 4 or 6 genotype scores

[c] n = 43

**Supplementary Table S16:** The *COL5A1* rs12722 (T/C), *COL11A1* rs3753841 (T/C), *COL11A1* rs1676486 (T/C) and *COL11A2* rs1799907 (A/T) genotype score effects on the general characteristics, Beighton score and sit and reach measurements of participants with a history of an uninjured non-dominant leg, as well as those with a history of non-dominant lower limb injury and/or treatment.

| | **Genotype Score** | | | |
|---|---|---|---|---|
| | **0** | **2** | **4 or 6** | **P-value** |
| | n = 51 | n = 58 | n = 20 | |
| **Sex** (% male) | 58.8 | 56.9 | 65.0 | 0.817 |
| **Age** (years) | 27.0 (25.0; 32.0) | 28.0 (23.8; 33.0) | 25.0 (23.3; 29.5) | 0.296 |
| **Height** (cm) | 175.4 ± 10.3 | 173.1 ± 9.0 | 177.1 ± 10.4 | 0.221 |
| **Body mass** (kg) | 74.8 ± 13.4 | 71.8 ± 13.5 | 72.5 ± 11.3 | 0.497 |
| **BMI** ($kg/m^2$) | 23.8 (21.6; 26.0) | 23.7 (21.9; 25.7) | 23.7 (21.1; 24.3) | 0.539 |
| **Flexibility Training** (% Yes) | 62.7 | 51.7 | 50.0 | 0.436 |
| **Beighton Score** | 2.0 (0.0; 4.0) | 1.0 (0.0; 5.0) | 1.0 (0.0; 3.0) | 0.140 |
| **Sit and Reach** (cm) | 42.4 ± 11.8 [a] | 43.5 ± 10.2 [b] | 41.0 ± 10.7 | 0.478 |

Sex and flexibility training are reported as percentages, while the the remaining continuous variables are reported as average ± standard deviation or median (IQR)

Participants received a genotype score of 2 for each *COL5A1* rs12722 CC, *COL11A1* rs3753841 CC, *COL11A1* rs1676486 TT and *COL12A1* rs1799907 AA genotypes and 0 for all other genotypes, so that the genotype scores ranged from 0 to 8

None of the participants had a genotype score of 8 and the genotype scores of 4 (n = 15) and 6 (n = 5) combined for the analysis

Internal and external rotation, as well as slack were not measured in the participants with a history of non-domiant lower limb injury and/or treatment using the Robotic Knee Testing (RKT) device

BMI, body mass index

[a] n = 49; [b] n = 56

**Supplementary Table S17:** The *COL5A1* rs12722 (T/C), *COL11A1* rs3753841 (T/C), *COL11A1* rs1676486 (T/C) and *COL11A2* rs1799907 (A/T) genotype score effects on the (i) passive and active genu recurvatum measurements, and (ii) anterior (Ant) and posterior (Post) tibial translation, active and maximum (Max) displacement as well as compliance index measured using the KT-1000 arthrometer of participants with a history of an uninjured non-dominant leg, as well as those with a history of non-dominant lower limb injury and/or treatment.

| | Genotype Score | | | Global P-value | Mutiple Comparisons P-value |
|---|---|---|---|---|---|
| | 0 | 2 | 4 or 6 | | |
| **Genu Recurvatum** [a] | n = 51 | n = 58 | n = 20 | | |
| **Passive** (°) | 176.0 (173.0; 179.0) | 177.0 (173.0; 182.0) | 179.0 (176.3; 181.0) | 0.057 | 0.036 [b] |
| **Active** (°) | 173.8 ± 4.8 | 175.0 ± 6.0 | 176.9 ± 5.3 | 0.093 | 0.032 [c] |
| **Anterior-Posterior Tibial Translation** | n = 51 | n = 58 | n = 20 | | |
| ▪ **133 N Ant Translation** (mm) | 7.0 (5.0; 9.5) | 7.5 (6.0; 9.0) | 7.0 (6.0; 8.0) | 0.750 | 1.000 [b] |
| ▪ **133 N Post Translation** (mm) | 4.0 (3.0; 5.5) | 4.0 (3.0; 5.8) [d] | 3.5 (2.7; 5.0) | 0.460 | 0.614 [b] |
| ▪ **Compliance Index** (mm) | 4.5 (3.0; 6.9) | 4.5 (3.5; 6.6) | 4.5 (4.0; 6.0) | 0.895 | 1.000 [b] |
| ▪ **Active Displacement** (mm) | 5.2 ± 2.6 | 5.1 ± 2.9 [c] | 4.6 ± 2.4 | 0.705 | 0.477 [c] |
| ▪ **Max Displacement** (mm) | 7.5 (5.0; 10.5) | 8.0 (6.0; 10.1) | 7.5 (5.3; 9.0) | 0.749 | 1.000 [b] |

Variables are reported as average ± standard deviation or median (IQR)

Participants received a genotype score of 2 for each *COL5A1* rs12722 CC, *COL11A1* rs3753841 CC, *COL11A1* rs1676486 TT and *COL12A1* rs1799907 AA genotypes and 0 for all other genotypes, so that the genotype scores ranged from 0 to 8

None of the participants had a genotype score of 8 and the genotype scores of 4 (n = 15) and 6 (n = 5) combined for the analysis

Internal and external rotation, as well as slack were not measured in the participants with a history of non-domiant lower limb injury and/or treatment using the Robotic Knee Testing (RKT) device

Compliance index = 133 N - 67 N Anterior Translation

[a] Lower degree values for genu recurvatum indicates a greater amount of hyperextension

[b] Dunn’s multiple comparisons test respectively of the 0 versus 4 or 6 genotype scores

[c] Test for linear trend

[d] n = 57

**Supplementary Table S18:** Summary of multiple linear regression model for active genu recurvatum of participants with a history of an uninjured non-dominant leg.

| **Passive Genu Recurvatum** | **Unstandardised coefficient (β)** | **95% CI for β** | **SE β** | **P-Value** |
|---|---|---|---|---|
| Constant | 174.7 | 166.2 to 183.1 | 4.27 | <0.001 |
| Sex (male) | 4.48 | 1.77 to 7.19 | 1.37 | 0.001 |
| Age (years) | 0.20 | -0.01 to 0.40 | 0.10 | 0.060 |
| Body Mass (kg) | -0.12 | -0.23 to -0.02 | 0.05 | 0.026 |
| Genotype Score (2) | 1.51 | -0.84 to 3.87 | 1.19 | 0.206 |
| Genotype Score (4 or 6) | 2.94 | -0.17 to 6.03 | 1.56 | 0.063 |
| $R^2 = 0.18$ and Adjusted $R^2 = 0.14$ | | | | |

CI = confidence interval; SE β = standard error of the coefficient; $R^2$ = coefficient of determination

Units are shown in parentheses

*COL5A1*, *COL11A1*, *COL11A2* genotyping score coding: 0, 2 and 4 or 6

**Supplementary Table S19:** Summary of multiple linear regression model for passive genu recurvatum of participants with a history of an uninjured non-dominant leg, as well as those with a history of non-dominant lower limb injury and/or treatment.

| **Passive Genu Recurvatum** | **Unstandardised coefficient (β)** | **95% CI for β** | **SE β** | **P-Value** |
|---|---|---|---|---|
| Constant | 177.9 | 170.9 to 184.8 | 3.52 | <0.001 |
| Sex (male) | 4.94 | 2.55 to 7.34 | 1.21 | <0.001 |
| Age (years) | 0.15 | -0.01 to 0.30 | 0.08 | 0.063 |
| Body Mass (kg) | -0.12 | -0.21 to -0.03 | 0.05 | 0.009 |
| Genotype Score (2) | 1.03 | -0.98 to 3.04 | 1.03 | 0.311 |
| Genotype Score (4 or 6) | 3.40 | 0.63 to 6.17 | 1.40 | 0.017 |
| $R^2$ = 0.18 and Adjusted $R^2$ = 0.15 | | | | |

CI = confidence interval; SE β = standard error of the coefficient; $R^2$ = coefficient of determination

Units are shown in parentheses

*COL5A1*, *COL11A1*, *COL11A2* genotyping score coding: 0, 2 and 4 or 6

**Supplementary Table S20:** The *COL5A1* rs12722 (T/C), *COL11A1* rs3753841 (T/C), *COL11A1* rs1676486 (T/C) and *COL11A2* rs1799907 (A/T) genotype score effects on the absolute change in the length of the anterior bundle of the ACL (aACL), posterior bundle of the ACL (pACL), anterior bundle of the PCL (aPCL), posterior bundle of the PCL (pPCL), anterior bundle of the superficial layer of the MCL (aMCL), inferior bundle of the superficial layer of the MCL (iMCL), anterior bundle of the deep layer of the MCL (aDMCL), posterior bundle of the deep layer of the MCL (pDMCL), posterior bundle of the superficial layer of the MCL (pMCL) and the LCL of the non-dominant leg.

| | **Genotype Score** | | | |
|---|---|---|---|---|
| | **0** | **2** | **4 or 6** | **P-Value** |
| | n = 31 | n = 33 | n = 12 | |
| **aACL** (mm) | 1.2 (0.8; 1.6) | 1.3 (0.9; 1.5) | 1.0 (0.7; 1.6) | 0.655 |
| **pACL** (mm) | 2.7 ± 1.4 | 3.0 ± 1.2 | 3.0 ± 1.4 | 0.925 |
| **aPCL** (mm) | 2.2 (1.3; 2.9) | 1.5 (0.9; 3.1) | 1.5 (1.3; 3.2) | 0.480 |
| **pPCL** (mm) | 1.4 (0.7; 2.9) | 1.3 (1.0; 2.1) | 1.4 (0.6; 2.5) | 0.929 |
| **aMCL** (mm) | 3.8 (3.0; 5.0) | 3.5 (3.0; 4.8) | 3.7 (2.2; 4.6) | 0.900 |
| **iMCL** (mm) | 3.3 (2.8; 4.2) | 3.1 (2.7; 4.4) | 3.2 (2.0; 4.3) | 0.838 |
| **aDMCL** (mm) | 6.1 (4.6; 7.6) | 5.4 (4.9; 7.5) | 5.5 (4.7; 7.5) | 0.875 |
| **pDMCL** (mm) | 6.8 (5.3; 7.9) | 6.1 (5.2; 8.3) | 6.0 (5.5; 8.1) | 0.995 |
| **pMCL** (mm) | 4.1 ± 1.6 | 4.1 ± 1.4 | 3.9 ± 1.3 | 0.845 |
| **LCL** (mm) | 1.1 (0.9; 1.8) | 1.3 (0.8; 1.7) | 1.1 (0.7; 2.0) | 0.919 |

Values are expressed as either average ± standard deviation or median (IQR)

Participants received a genotype score of 2 for each *COL5A1* rs12722 CC, *COL11A1* rs3753841 CC, *COL11A1* rs1676486 TT and *COL12A1* rs1799907 AA genotypes and 0 for all other genotypes, so that the genotype scores ranged from 0 to 8

None of the participants had a genotype score of 8 and the genotype scores of 4 (n = 10) and 6 (n = 2) combined for the analysis

**Supplementary Material References**

Brown, J. C., C. J. Miller, M. P. Schwellnus, and M. Collins. 2011. 'Range of motion measurements diverge with increasing age for COL5A1 genotypes', *Scand J Med Sci Sports*, 21: e266-72.

Hay, M., J. Patricios, M. Collins, A. Branfield, J. Cook, C. J. Handley, A. V. September, M. Posthumus, and M. Collins. 2013. 'Association of type XI collagen genes with chronic Achilles tendinopathy in independent populations from South Africa and Australia', *Br J Sports Med*, 47: 569-74.

Lim, S. T., C. S. Kim, W. N. Kim, and S. K. Min. 2015. 'The COL5A1 genotype is associated with range of motion', *J Exerc Nutrition Biochem*, 19: 49-53.
